# Synergistic lignin degradation between *Phanerochaete chrysosporium* and Fenton chemistry is mediated through iron cycling and ligninolytic enzyme induction.

**DOI:** 10.1016/j.scitotenv.2023.166767

**Published:** 2023-12-20

**Authors:** Julian J.A. van der Made, Elizabeth A. Landis, Griffin T. Deans, Ruby A. Lai, Kartik Chandran

**Affiliations:** a Department of Earth and Environmental Engineering, Columbia University, New York, NY, USA; b Department of Civil and Environmental Engineering, Stanford University, Palo Alto, CA, USA

**Keywords:** Lignin, Fenton, *Phanerochaete chrysosporium*, White rot, Ligninolytic enzymes, Iron redox cycling

## Abstract

Removal of recalcitrant lignin from wastewater remains a critical bottleneck in multiple aspects relating to microbial carbon cycling ranging from incomplete treatment of biosolids during wastewater treatment to limited conversion of biomass feedstock to biofuels. Based on previous studies showing that the white rot fungus *Phanerochaete chrysosporium* and Fenton chemistry synergistically degrade lignin, we sought to determine optimum levels of Fenton addition and the mechanisms underlying this synergy. We tested the extent of degradation of lignin under different ratios of Fenton reagents and found that relatively low levels of H_2_O_2_ and Fe(II) enhanced fungal lignin degradation, achieving 80.4 ± 1.61 % lignin degradation at 1.5 mM H_2_O_2_ and 0.3 mM Fe(II). Using a combination of whole-transcriptome sequencing and iron speciation assays, we determined that at these concentrations, Fenton chemistry induced the upregulation of 80 differentially expressed genes in *P. ch* including several oxidative enzymes. This study underlines the importance of non-canonical, auxiliary lignin-degrading pathways in the synergy between white rot fungi and Fenton chemistry in lignin degradation. We also found that, relative to the abiotic control, *P. ch.* increases the availability of Fe(II) for the production of hydroxyl radicals in the Fenton reaction by recycling Fe(III) (p < 0.001), decreasing the Fe(II) inputs necessary for lignin degradation via the Fenton reaction.

## Highlights

• *P. chrysosporium* is combined with Fenton oxidation to optimize lignin degradation.
• *P. ch.* plus Fenton synergistically degrade lignin over individual processes.
• Fenton chemistry promotes transcription of lignin-degrading enzymes in *P. ch.*
• *P. ch.* cycles iron, lowering iron inputs needed for Fenton oxidation.

## Graphical abstract

**Figure:**
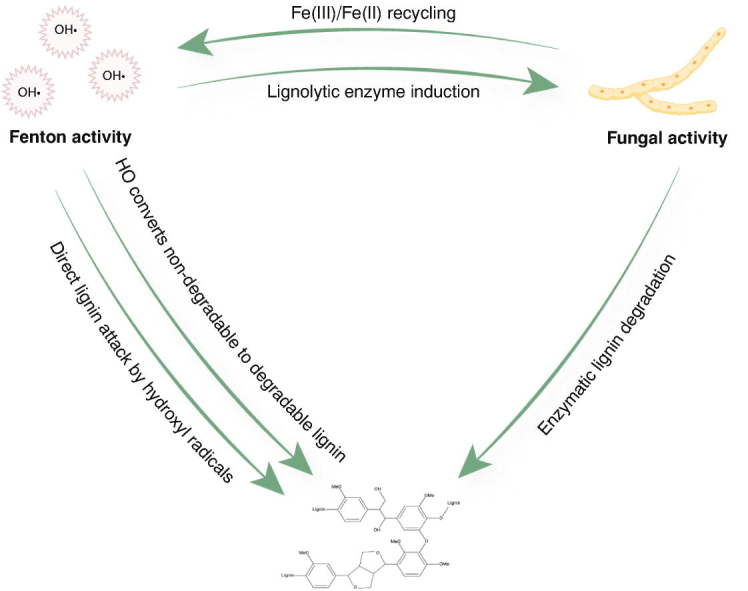
Conceptual schematic of synergistic relationship between white rot fungi and Fenton chemistry. Green arrows indicate promotion. We demonstrate that Fenton chemistry (top left) promotes ligninolytic enzyme induction and directly contributes to the degradation of lignin.

## 1 Introduction

The limited biotransformation of recalcitrant organic compounds is a technological bottleneck in water treatment and waste resource recovery. A large portion of the heterogeneous mix of organic compounds that make up wastewater and excess sludge consists of lignocellulose (Hu et al., 2016; Liu and Smith, 2022). Lignin hampers carbon recovery from lignocellulosic material by encrusting and preventing the valorization of hemicellulose and cellulose (Hu et al., 2016; Liu and Smith, 2022). Recently-developed technologies to delignify biomass or remove aromatic pollutants include thermal pre-treatment and advanced oxidation processes such as Fenton-based treatments. Although these technologies have promising performance, they require costly energy and/or chemical inputs and can release inhibitory byproducts from lignin or additional waste streams (Del Álamo et al., 2022; Teixeira et al., 2014). For example, conventional Fenton processes require the input of unstable and expensive homogeneous solutions of ferrous iron and can produce iron sludge due to the precipitation of ferric iron, which is difficult to separate and recover (Zhang et al., 2019; Bello et al., 2019).

One especially promising but unrealized avenue for the engineered biotransformation of recalcitrant organic compounds is the inclusion of white rot fungi (WRF) or their oxidative enzymes (Kameshwar and Qin, 2017; Janusz et al., 2017). These enzymes, including manganese peroxidase, lignin peroxidase, and laccase, have been shown to non-specifically transform a variety of recalcitrant organic compounds including lignin (Del Álamo et al., 2022; Barber et al., 2020). Additionally, since WRF can fully mineralize lignin, there is comparatively minimal formation of byproducts inhibitory to downstream processes, such as anaerobic digestion (Teixeira et al., 2014).

However, the biological potential of WRF has not been translated to engineered bioprocess reactors due to the operational challenges that fungi present (Sankaran et al., 2010; More et al., 2010; Mir-Tutusaus et al., 2018). On one hand, fungal populations can be difficult to maintain. They can require nutrient supplementation and are sensitive to shear stress and microbial competition (Mir-Tutusaus et al., 2018; Mir-Tutusaus et al., 2016). On the other hand, excessive growth of fungal biomass in bioreactors can be problematic; excess mycelia can wrap around impellers, cause blockages in influent and effluent lines, and increase viscosity, thereby limiting mass transfer (Moreira et al., 2003).

Even if fungal biomass is maintained and managed in an engineered process, its ligninolytic activity can be unstable (Singh and Chen, 2008; Moreira et al., 2003). As part of its secondary metabolism, ligninolytic activity in WRF is governed by a complex set of nutritional, physiological, and environmental conditions and involves the production of a battery of intracellular and extracellular enzymes and redox-mediating metabolites which are not fully understood in terms of their mechanisms or their interactions (Singh and Chen, 2008; Moreira et al., 2003; Mir-Tutusaus et al., 2018). To date, such operational challenges and knowledge gaps have prevented the adaptation of fungal bioprocesses in all but a few small-scale cases dealing with industry effluents. In order to realize the promise and potential of white-rot fungal metabolism into engineered bioprocess technologies, as a first step, improved characterization of their metabolic capabilities and biokinetics is needed.

**Fig. 1:**
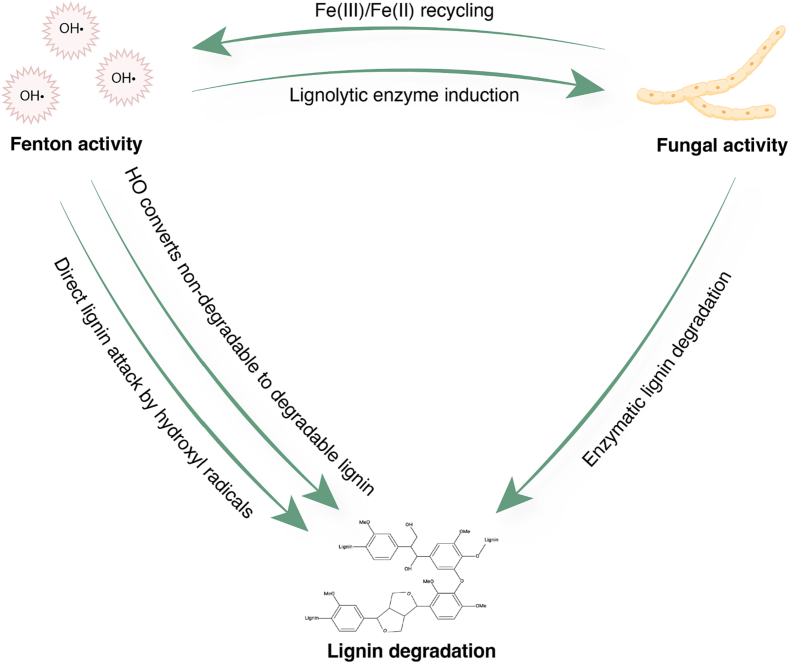
Conceptual diagram of synergistic relationship between white rot fungi and Fenton chemistry. Green arrows indicate promotion. For example, we propose that Fenton chemistry (top left) promotes ligninolytic enzyme induction and directly contributes to the degradation of lignin.

White rot fungal enzymes and Fenton chemistry have been shown to synergistically degrade lignin, and previous studies have attributed this synergy to direct lignin oxidation and modification by Fenton chemistry, induction of fungal ligninolytic enzymes LiP, MnP, and laccase, and an increase in H_2_O_2_, which is a required co-substrate for certain lignin-degrading enzymes (Hou et al., 2020; Merino et al., 2020). In this study, we further elucidate the mechanisms underpinning the synergy between Fenton chemistry and WRF and propose that these two technologies may be complementary in more dimensions than previously understood (Fig. 1), utilizing the model WRF *Phanerochaete chrysosporium.*

We hypothesized two important mechanisms for synergistic increases in lignin degradation which had not been previously investigated. First, although previous studies utilized qPCR to target the canonical lignin degrading enzymes in *P. ch*: LiP and MnP (Hou et al., 2020), we expected a broader transcriptional response to Fenton than just the upregulation of LiP and MnP, given that under ligninolytic conditions *P. ch.* has been shown to upregulate a wide variety of genes including several other oxidative and auxiliary enzymes (Wymelenberg et al., 2009) Second, we hypothesized that *P. ch.* could reduce the quantity of Fenton reagents needed for effective compound degradation because certain wood-rotting fungi have been shown to produce various low molecular weight Fe(III)-reducing compounds and iron-solubilizing carboxylic acids, such as phenolate-derivative compounds and oxalic acid (Gómez-Toribio et al., 2009; Arantes et al., 2011). Thus, *P. ch.* could solubilize Fe(III) and reduce it to Fe(II) (Fig. 1). This would be a significant advantage to combining these technologies since the cost of chemical inputs and the generation of iron sludge are major limitations to Fenton processes (Zhang et al., 2019; Bello et al., 2019).

In this study, we utilized soluble alkali lignin as a model organic compound to represent soluble and insoluble lignin (Su et al., 2018). We measured its degradation across a range of Fenton compound concentrations both with and without fungi in order to confirm the synergistic interaction between the two technologies and identify the concentrations of Fenton reagents that optimize lignin degradation in the combined system. Using whole-transcriptome sequencing and iron speciation assays, we identified potential mechanisms underlying this synergy. Additionally, we tested the effects of Fenton compounds on the biomass formation of *P. ch*. and investigated the relationship between biomass and lignin degradation. Taken together, our results elucidate mechanisms underlying the enzymatic degradation of lignin by *P. ch.* in synergy with Fenton chemistry and suggest a framework for how Fenton chemistry might be incorporated into ligninolytic bioprocesses utilizing WRF*.*

## 2 Materials and methods

### 2.1 Fungal inoculum and culture conditions

*Phanerochaete chrysosporium* (ATCC 24725) was cultured on malt extract agar (MEA) containing 2 % (w/v) malt extract and 2 % (w/v) agar for 5 days at 39 °C. A spore suspension was prepared by flooding the Petri dish with 0.9 % (w/v) sterile saline with 0.05 % (v/v) Tween 80 and dislodging the spores using a Drigalski spatula. The spore suspension was filtered through sterilized cheesecloth and the absorbance at 650 nm was measured and adjusted to 0.5 using sterile saline and inoculated into Kirk and Tien (K&T) medium as outlined in (Tien and Kent Kirk, 1988), except that the dimethylsuccinate buffer was replaced by 10 mM citrate buffer (pH = 4.5). Alkali lignin (TCI America, L0082) was added to the medium at 500 mg/L. Batch incubations contained 1.8 mL of medium and 0.2 mL of spore suspension in 14 mL plastic culture tubes with the snap cap in the aerobic position. Incubation was conducted at 39 °C on a rotary shaker at 250 rpm for 10 days.

### 2.2 Effect of Fenton reagents on fungal growth and lignin degradation

For batch tests evaluating the effect of different concentrations of Fenton reagents on lignin degradation, the medium contained 10 g/L glucose and 0.2 g/L ammonium tartrate, as per (Tien and Kent Kirk, 1988). A 100 mM ferrous sulfate solution in 10 mM citrate buffer (pH = 4.5) and a 3 % hydrogen peroxide solution were used to prepare the Fenton reagents at differing concentrations. Fenton combinations of Fe and H_2_O_2_ were implemented with a 5 × 5 factorial design with [Fe] = 0, 0.1, 0.3, 0.5, and 1 mM and [H_2_O_2_] = 0, 0.5, 1.5, 5, 10, 20 mM. For all combinations of [Fe] and [H_2_O_2_] and controls, N = 5. Iron was dosed once on day 0 of the incubation and H_2_O_2_was dosed every 24 h. The rationale behind this design was that unlike H_2_O_2_, which gets consumed in Fenton reactions, Fe(II) gets oxidized to Fe(III), and Fe(II) can subsequently be regenerated from Fe(III) either through a reaction with additional H_2_O_2_ or through fungal activity. Fungal biomass was measured on day 10 after separation from the liquid medium and residual lignin concentrations were measured in the liquid portion. Fungal biomass was dried at 65 °C for 24 h to determine dry weight.

### 2.3 Fe(II) production and hydroxyl radical production

To evaluate the ability of *P. ch.* to produce Fe(II) from Fe(III) and to produce hydroxyl radicals, *P. ch.* was grown in K&T medium as described in Section 2.1 without the addition of any Fenton reagents. After 5 days of growth, reagents for each assay were added and the cultures were incubated for an additional 30 min before measuring absorbance.

To measure production of Fe(II) from Fe(III), 1.5 mM 1,10-phenanthroline and 0.3 mM Fe(III) (final concentrations) were added to the incubations from a freshly prepared stock solution of 100 mM ferric chloride in 10 mM citrate buffer (pH = 4.5) (Gómez-Toribio et al., 2009). The concentration of Fe(II) produced in the culture supernatant was measured by absorbance at 510 nm. Other assays for Fe(II) are also commonly used, most notably the Ferrozine assay. Herein, the phenanthroline method as presented in the Standard Methods for the Examination of Water and Wastewater (3500-Fe B. Phenanthroline Method), was chosen.

For experiments evaluating hydroxyl radical production, thiobarbituric acid reactive substances (TBARS) production from 2-deoxyribose was used as a proxy (Gómez-Toribio et al., 2009); 2.8 mM 2-deoxyribose and 1.5 mM of H_2_O_2_ (final concentrations) were added and TBARS was measured as follows: 0.5 mL of 2.8 % trichloroacetic acid and 0.5 mL of 1 % (w/v) thiobarbituric acid in 50 mM NaOH was added to 1 mL of supernatant. The mixture was heated for 20 min at 100 °C and the absorbance was measured at 532 nm.

### 2.4 RNA sample preparation and sequencing

We employed whole genome transcriptomics to understand the effect of Fenton chemistry on global gene expression in *P.ch*. batch cultures (2.2, 2.3). RNA was extracted from fungal biomass following the daily addition of Fenton reagents at the optimum concentrations for lignin degradation (1.5 mM H_2_O_2_, and 0.3 mM Fe(II)). These trials were conducted in 2 mL shaking tubes, in identical conditions to the batch tests of lignin degradation, except that the fungal biomass was harvested at day 5 rather than day 10 in order to capture a profile of fungal gene expression earlier in the degradation of lignin. Fungal biomass was removed 1.5 h after the addition of Fenton reagents, suspended in 3 mL of RNAlater and frozen at −80 °C. RNA was extracted after samples were thawed, and fungal biomass was pulverized by grinding in liquid nitrogen. Total RNA was extracted using Qiagen's RNeasy PowerSoil Total RNA kit, following the manufacturer's instructions. RNA samples were checked for quality using a NanoDrop (Thermo Fisher Scientific, USA) and were sequenced at Genewiz (Shanghai, China) with 150 bp paired-end sequencing on Illumina HiSeq with poly A selection.

### 2.5 Bioinformatics

Reads were filtered using Trimmomatic (Bolger et al., 2014) using a minimum phred score of 33 and the following parameters: ILLUMINACLIP:TruSeq3-PE.fa:2:30:10 LEADING:3 TRAILING:3 SLIDINGWINDOW:4:15 MINLEN:36. Pairs where both reads survived were retained for downstream processing. Hisat2 (Kim et al., 2019) was used to align trimmed reads to the reference genome: *Phanerochaete chrysosporium* RP-78 v4.2 (Ohm et al., 2014), which was accessed through JGI. HTSeq was employed for creating count tables. EdgeR (Robinson et al., 2010) was used to find DE genes, using the tagwise dispersion method and significance values derived from FDR-corrected P values of Fisher's exact tests. A gene was considered differentially expressed if the log_2_-fold change was >2 and had a FDR-corrected p-value of <0.01. We contend that the stringent criteria used for classifying a gene as differentially expressed does not warrant validation with qPCR, especially given the close concordance between RNA-seq and qPCR observed in other studies (Coenye, 2021). Gene ontology (GO) enrichments were assigned using the R package TopGO (Alexa and Rahnenfuhrer, 2023); and the results were limited to the “molecular function” family of terms. TopGO was run with a node size of 10 and Kolmogorov Smirnov tests using the “classic” method were used to obtain P values. P values <0.01 were considered enriched. These enriched GO terms were then simplified using Revigo (Supek et al., 2011), which clustered by semantic similarity, referencing the whole Uniprot database and employing the SimRel semantic similarity measure. Kyoto Encyclopedia of Genes and Genomes (KEGG) annotations were assigned using KofamKOALA (Aramaki et al., 2020) and gene set enrichment analysis for KEGG pathways and modules was conducted on the log2-fold changes of the full set of genes using the gseKEGG function of ClusterProfiler (Wu et al., 2021).

### 2.6 Lignin quantification

Residual lignin concentrations in the culture supernatant were measured using reverse-phase high-performance liquid chromatography (RP-HPLC) (Thermo Scientific Ultimate 3000). Samples were centrifuged at 10,000 ×*g* for 5 min and diluted using 0.1 % acetic acid in ultrapure water. Separation was performed on a Thermo Scientific Acclaim 300 C18 HPLC column (150 mm × 2.1 mm, particle size 3 μm). We used a 300 Å pore column due to the high molecular weight of alkali lignin, which can be >50 kDa (S. Liu et al., 2020). The flow rate was 0.3 mL/min, injection volume was 25 μL, and column temperature was held at 40 °C. The mobile phases were 0.1 % acetic acid in ultrapure water (solvent A) and acetonitrile (solvent B). The gradient started at 15 % B for 4 min followed by a 1 min gradient to 75 % B, which was held for 3 min, followed by a flush of 90 % B for 2 min. The column was re-equilibrated for 10 min in between each sample. Peaks were monitored using a DAD detector at 280 nm and integrated using the Chromeleon software and a custom Python script. The method was validated by running all components of the medium individually to ensure proper separation. A standard curve relating peak area to lignin concentration was constructed by running the initial medium with varying concentrations of alkali lignin (0–500 mg/L) under the same HPLC conditions. All samples and standards were run in duplicate.

## 3 Results and discussion

### 3.1 Confirming and optimizing synergy between *P. ch.* and Fenton chemistry

**Fig. 2:**
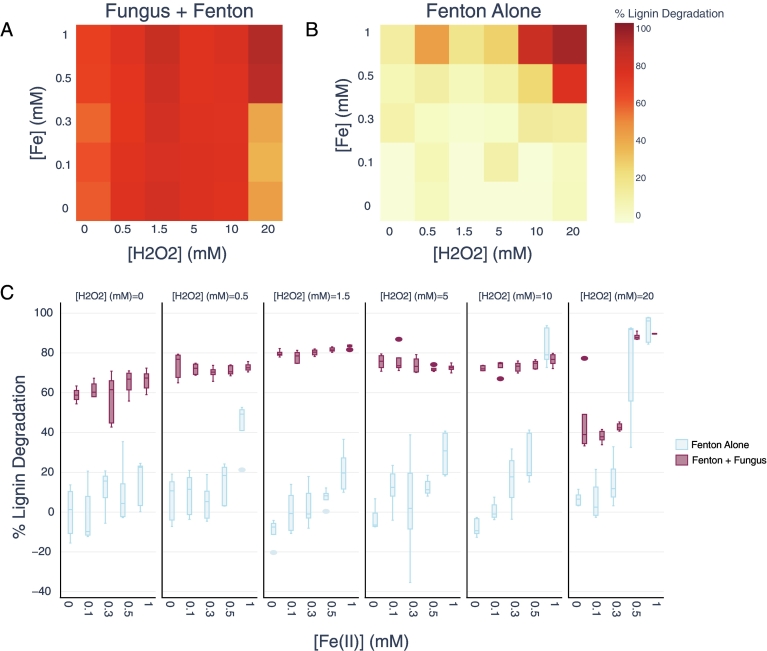
Lignin degradation is enhanced by combining *P. ch.* and Fenton chemistry, with an H_2_O_2_ optimum of 1.5 mM. In the absence of *P. ch.*, lignin is only degraded at the highest concentrations of Fenton reagents. A) Lignin degradation in the combined treatment B) Lignin degradation with only Fenton chemistry C) Same as A and B, but as box plots showing interquartile ranges and median values. Alkali lignin concentrations were measured after 10-day incubations, and for each treatment N = 5.

We found that *P. ch.* alone degraded 58.8 ± 3.67 % of lignin after 10 days, and that Fenton chemistry alone degraded 92.3 ± 6.69 %, but only at the highest inputs of Fenton reagents ([H_2_O_2_] = 20 mM and [Fe(II)] = 1 mM). The dosing concentration of H_2_O_2_ was a significant driver of lignin degradation (Fig. 2) both in the *P. ch.* + Fenton treatments (F(4) = 63, p < 0.001) and in the Fenton alone treatments (F(4) = 29, p < 0.001). Lignin degradation in the combined treatment was highest at [H_2_O_2_] = 1.5 mM, declining at higher concentrations of H_2_O_2_, and significantly different than any other H_2_O_2_-dosing concentration (Tukey p < 0.001, see supplementary information for full table).

**Fig. 3:**
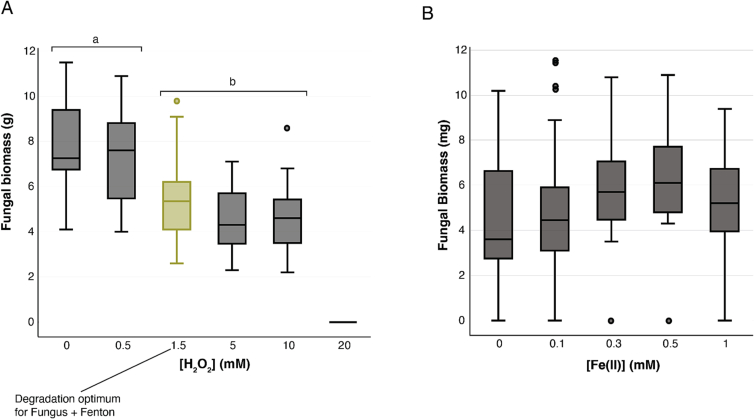
Fungal biomass declines for H2O2 concentrations at or above 1.5 mM (the degradation optimum). A) Fungal biomass across all five iron treatments for each concentration of H2O2 tested (F(4) = 76.3, ANOVA p < 0.001); N = 5 [Fe(II)] conditions and N = 5 replicates, so each box and whisker plot represents *N* = 25 samples, showing interquartile ranges and median values. Labels represent significant differences between groups (Tukey's P each <0.001 between significant comparisons). B) Fungal biomass across all five H2O2 treatments for each concentration of Fe(II) tested. N = 5 [H2O2] conditions and N = 5 replicates, so each box and whisker plot represents N = 25 samples, showing interquartile ranges and median values). Iron did not significantly affect fungal biomass (F(4) = 1.56, p = 0.19).

At this optimal H_2_O_2_ concentration of 1.5 mM, a synergistic effect between fungal activity and Fenton chemistry toward lignin degradation was observed. To illustrate, at [H_2_O_2_] = 1.5 mM and [Fe(II)] = 0.3 mM, there was negligible lignin degradation (−0.95 ± 9.89 %) by Fenton activity alone, and *P. ch.* degraded 58.8 ± 3.67 %. Together, at these same concentrations of Fenton reagents, 80.4 ± 1.61 % of the lignin was degraded (Fig. 3A).

**Fig. 4:**
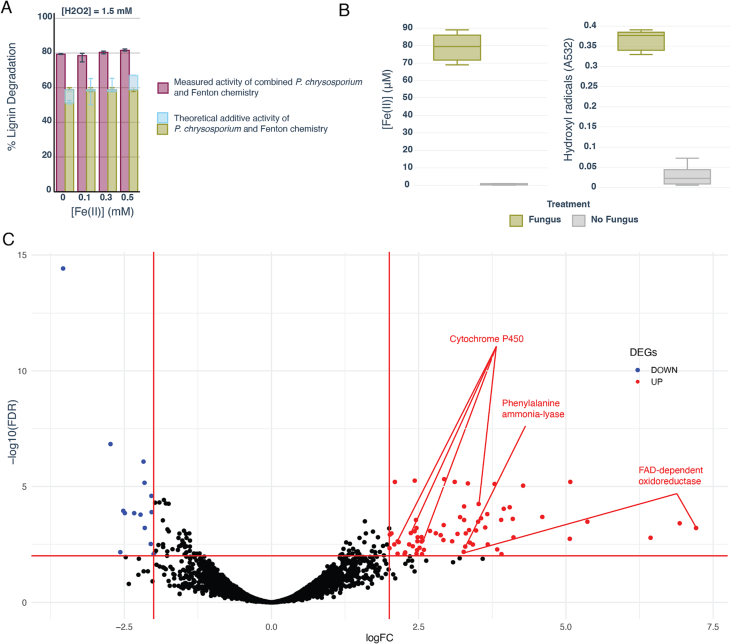
Fe(III)/Fe(II) cycling activity observed in *P. ch.* and induction of lignin-degrading genes by Fenton chemistry could explain synergistic lignin degradation. A) Comparison of measured combined lignin degradation activity of *P. ch.* and Fenton chemistry to the theoretical additive activity of *P. ch.* and Fenton chemistry alone. B) Fe(II) and hydroxyl radicals produced in the presence of 0.3 mM Fe(III) in 30 min in *P. ch.* cultures versus uninoculated media. C) Individual genes which are differentially expressed according to a Fisher's exact test. Red lines indicate cutoffs for significant genes discussed in the text (log2-fold changes>2 and p < 0.01).

This set of experiments also pointed to the range over which *P. ch.* produces biomass under different amounts of Fenton reagents, with *P. ch.* biomass production declining at 1.5 mM of H_2_O_2_ (Tukey p < 0.001) and producing no biomass at 20 mM (Fig. 4A). Iron dosing concentrations did not significantly affect fungal biomass (F(4) = 1.56, p = 0.19) (Fig. 4B). Interestingly, fungal biomass had a negative correlation with lignin degradation (r(121) = −0.42, p < 0.001), implying that over the range of Fenton reagent concentrations tested, there was a tradeoff between production of biomass and degradation of lignin. This is supported by theoretical assumptions and by experimental evidence (Zheng et al., 2020) which posit that because oxidative enzymes are metabolically expensive, fungi trade off growth with enzyme production. The finding that fungal biomass and lignin degradation do not have the same optimum is promising for the inclusion of WRF in bioreactors since excess fungal biomass can cause operational difficulties in a bioreactor setting (Couto and Toca-Herrera, 2007; Espinosa-Ortiz et al., 2015).

### 3.2 Mechanisms underlying synergy between *P. ch.* and Fenton chemistry

#### 3.2.1 *P. ch.* increases the availability of Fe(II)

The dosing concentration of iron was highly significant in the Fenton alone treatments (F(4) = 80, p < 0.001) and statistically not significant in the combined *P. ch.* + Fenton treatments (F(4) = 40, p = 0.062). Notably, the calculations did not include the [H_2_O_2_] = 20 mM for the *P. ch.* + Fenton treatments, because *P. ch.* did not persist at this concentration of H_2_O_2_. When [H_2_O_2_] = 20 mM and *P. ch.* was no longer present, Fe(II) became a significant driver of lignin degradation in the *P. ch.* + Fenton treatment (F(4) = 42, p < 0.001). Together, these results suggest that iron availability is not a limiting factor for lignin degradation in the presence of *P. ch.*

We hypothesized that *P. ch.* activity could be increasing the availability of Fe(II) by solubilizing iron and recycling Fe(III) for the production of hydroxyl radicals in the Fenton reaction. When Fe(III)-reducing activity was measured in 5-day-old cultures of *P. ch,* the fungal cultures produced 79.5 ± 8.32 μM Fe(II) in the span of 30 min, significantly (t(8) = 21.04, p < 0.001) more than the negligible amounts of Fe(II) (0.56 ± 0.42 μM) that were produced in the uninoculated samples (Fig. 4B). The inoculated samples also had significantly (t(8) = 19.87, p < 0.001) higher levels of hydroxyl radical production than the uninoculated samples (Fig. 4B). These data suggest that *P. ch.* aids hydroxyl radical production in the Fenton reaction by increasing Fe(II) availability through reduction and solubilization of iron.

In the conventional homogeneous Fenton reaction, the regeneration of Fe(II) from Fe(III) is much slower than its consumption (Zhang et al., 2019). Additionally, Fe(III) precipitates at pH values higher than 3 or in the absence of suitable chelators, leading to iron sludge formation (Bello et al., 2019; Zhang et al., 2019). These challenges necessitate the usage of higher Fe(II) concentrations in engineered Fenton processes to achieve desired degradation rates (Bello et al., 2019; Zhang et al., 2019). However, our data suggest that Fe(II)/Fe(III) cycling by *P. ch.* promotes efficient hydroxyl radical production even at low total iron concentrations, which could relieve the requirement for high iron inputs in Fenton processes.

#### 3.2.2 Fenton chemistry induces auxiliary pathways to lignin degradation in *P. ch.*

Another mechanism that could explain synergistic lignin degradation is the induction of fungal ligninolytic enzyme activity in the presence of reactive oxygen species. Hou et al. (2020) used RT-qPCR to measure the expression of the target enzymes lignin peroxidase (LiP) and manganese peroxidase (MnP) in *P. ch.* and found that expression was increased in the presence of electro-Fenton chemistry, presumably as a response to oxidative stress. Given that lignin degradation and modification is known to involve a suite of accessory enzymes beyond LiP and MnP (Kameshwar and Qin, 2017; Janusz et al., 2017), we utilized an untargeted approach to gain a broader understanding of changes in gene expression in *P. ch.* after sustained exposure to Fenton chemistry. We utilized whole-transcriptome sequencing to identify differentially expressed genes (DEGs) in cultures extracted RNA from 5-day-old *P. ch.* cultures with and without exposure to Fenton reagents ([H_2_O_2_] = 1.5 mM and [Fe(II)] = 0.3 mM). The goal was to get a global understanding of the transcriptomic response in *P. ch.* in response to Fenton chemistry to further elucidate the mechanistic underpinnings of the observed synergy. Due to the close concordance between RNA-seq and qPCR generally (Coenye, 2021) and the stringent criteria used in the study for classifying a gene as differentially expressed, qPCR validation of the RNA-seq results was not conducted. Based on these results, however, specific genes can be targeted with qPCR in future studies.

Contrary to our expectations, we did not find DEGs known to be directly involved in lignin-degrading enzyme systems (MnP or LiP). This does not exclude the possibility that at other timepoints, these enzymes may be differentially expressed in the presence of Fenton chemistry. We did, however, find 83 genes which were significantly (log2 fold-change >2, FDR-corrected p < 0.01) over-expressed in the Fenton treatment and 14 genes which were over-expressed in the control treatment (Fig. 3C). Additionally, we found 35 enriched molecular function GO terms, 20 enriched KEGG pathways, and 10 enriched KEGG modules (see supplementary materials for tables of enriched GO and KEGG terms). Among the GO terms, those correlating with oxidoreductases, iron ion binding and catalytic activity were the most over-represented. The enrichment of these particular GO categories generally supports our hypothesis that the induction of fungal enzymes is an important mechanism for the Fenton-*P. ch.* synergy, and also points to the importance of non-canonical oxidative enzymes besides MnP and LiP in lignin degradation.

The most differentially expressed individual gene was annotated as an FAD-dependent oxidoreductase (log2 fold-change +7.2 in the combined treatment, p < 0.001). FAD-dependent oxidoreductases have been shown to inhibit lignin re-polymerization by reducing and therefore stabilizing lignin-derived phenoxy radicals produced during oxidative degradation (Marzullo et al., 1995; Samejima and Eriksson, 1992; Ai et al., 2014). Without stabilization, these soluble phenoxy radicals can both re-polymerize and inactivate fungal LiP, decreasing lignin-degradation efficiency. A protein annotated as caffeoyl-coA *O*-methyltransferase was part of the core enrichment in the phenylpropanoid biosynthesis pathway (NES = 1.627, FDR-corrected p = 0.005). In *P. ch*., this protein converts toxic phenoxy by-products into non-toxic, methylated phenolic groups, thus preventing phenoxy radical repolymerization and protecting cells from toxic byproducts (Le and Kim, 2016).

In the combined treatment, we found evidence for upregulation of genes involved in the stabilization of the LiP enzyme: phenylalanine ammonia lyase (log2 fold-change +3.4, p < 0.001) and tryptophan biosynthesis from the chorismate module (NES = 2.04, FDR-corrected p = 0.0018). Phenylalanine ammonia lyase initiates the production of veratryl alcohol (VA) from phenylalanine (Kameshwar and Qin, 2017). VA plays a well-established role in the stabilization of lignin derivatives as well as the LiP enzyme (Harper et al., 1996; Marzullo et al., 1995). Tryptophan and its indole derivative have been shown to increase LiP activity in WRF by protecting LiP from inactivation in a similar fashion to VA (Collins et al., 1997).

Other pathways and genes associated with catabolism of lignin-derived aromatic compounds were enriched in the Fenton treatment, including styrene degradation (NES = 1.75, FDR-corrected p = 0.015). Although styrene was not present in our study, the enzymes in the core enrichment of this pathway participate in the degradation of low-molecular weight lignin fragments through phenylacetate, a known intermediate in the degradation of lignin (Zhu et al., 2017; Kameshwar and Qin, 2017) and a precursor to 4-hydroxy-phenylacetic acid, which is an Fe(III)-reducing compound produced by certain wood decay fungi (Arantes et al., 2011). Notably, four genes annotated as cytochrome P450 oxidoreductases were also significantly (log2 fold-change >2, p < 0.01; see supplementary materials for exact P values) upregulated. These intracellular enzymes are thought to be involved in demethylation and hydroxylation of lignin-derived aromatics (Wolf et al., 2022; Del Cerro et al., 2021).

Our analysis found differential expression of a suite of accessory enzymes involved in lignin degradation, but did not show upregulation of the canonical lignin-degrading enzymes LiP and MnP. Hou et al. (2020) previously found that LiP and MnP steadily increased over the course of 96 h after beginning electro-Fenton addition. Our experiments differed in that we exposed fungi to chemical Fenton reagents for 5 days via once-daily dosing of H_2_O_2_,whereas electro-Fenton supplies H_2_O_2_ continuously. It is likely that the addition of Fenton chemistry quickly increases the initial depolymerization of lignin (and the expression of LiP and MnP), producing higher quantities of lignin-derived aromatic compounds. Our results reinforce the role of non-canonical lignin-degrading machinery involving the stabilization of LiP, prevention of lignin re-polymerization, and catabolism of low-molecular weight lignin fragments.

The differential expression of these accessory enzymes may have other applications beyond lignin degradation. Cytochrome P450s in particular, which were up-regulated under Fenton addition, are well-documented as degraders of several micropollutants in wastewater and display wide substrate versatility (Mir-Tutusaus et al., 2018). In *P. ch.,* cytochrome P450s have been shown to play a role in the degradation of industrial pollutants such as polycyclic aromatic hydrocarbons (Syed et al., 2010), chlorinated dioxins (Chigu et al., 2010), and neonicotinoid insecticides (Wang et al., 2019), to name a few. For fungal processes aiming to remove micropollutants, Fenton chemistry may be useful for inducing the expression of these versatile fungal enzymes.

Engineered processes utilizing white-rot fungi hold immense potential for sustainable solutions in lignocellulosic biomass pre-treatment, enabling efficient conversion of biomass into value-added products. With the exciting prospect for increased applications of engineered fungal processes, recent studies have explored the combination of white-rot fungi with Fenton processes to enhance degradation. However, despite promising results, the underlying mechanisms driving this synergy and strategies for optimizing it are still relatively unexplored. In this study, we have addressed these gaps by demonstrating the iron cycling capabilities of *P. ch.* without the need for externally-added mediators and by investigating the comprehensive molecular response of *P. ch.* to Fenton chemistry. Through transcriptomic analysis, we have uncovered the upregulation of various oxidative and auxiliary enzymes under Fenton conditions. Notably, our findings differed from a previous study by Hou et al. (2020), as we did not observe increased expression of the canonical lignin-degrading enzymes MnP and LiP in the presence of Fenton chemistry. Instead, we highlight upregulated auxiliary pathways involved in stabilizing lignin-degrading metabolites and enzymes, preventing lignin re-polymerization, and breaking down low-molecular weight lignin fragments. These novel insights expand our understanding of non-canonical lignin degradation machinery of *P. ch.* in synergy with Fenton chemistry. By providing fundamental knowledge and insights, our study lays the groundwork for the development of engineered bioprocesses that integrate fungal-chemical transformations.

## 4 Conclusions

The addition of Fenton chemistry synergistically enhanced lignin degradation in *P. ch.* cultures from 58.8 % to 80.2 %. Notably, this study provides novel insight into the underlying mechanisms driving this synergy. Our results suggest that *P. ch.* stimulates Fenton chemistry by cycling Fe(II)/Fe(III) and upregulating enzymatic pathways that prevent lignin repolymerization, stabilize ligninolytic enzymes, and catabolize lignin-derived aromatics*.* These findings improve our understanding of the transformation of lignin by *P. ch.* and highlight the relevance of non-canonical auxiliary enzymes and metabolites for the efficient degradation of lignin in conjunction with Fenton chemistry. Overall, the integration of Fenton chemistry presents an exciting opportunity to advance the technology-readiness white-rot fungal bioprocesses for lignocellulosic biomass pretreatment. The insights gained from our study lay the foundation for further exploration and optimization of engineered fungal systems that efficiently degrade lignin in a sustainable and cost-effective manner.

## CRediT authorship contribution statement

**Julian J.A. van der Made:** Conceptualization, Data curation, Formal analysis, Investigation, Methodology, Project administration, Visualization, Writing – original draft, Writing – review & editing. **Elizabeth A. Landis:** Conceptualization, Data curation, Formal analysis, Investigation, Methodology, Project administration, Visualization, Writing – original draft, Writing – review & editing. **Griffin T. Deans:** Investigation, Methodology. **Ruby A. Lai:** Conceptualization, Funding acquisition, Supervision, Writing – review & editing. **Kartik Chandran:** Conceptualization, Funding acquisition, Resources, Supervision, Writing – review & editing.

## Declaration of competing interest

The authors declare the following financial interests/personal relationships which may be considered as potential competing interests: Kartik Chandran reports financial support was provided by Bill & Melinda Gates foundation. None.

## Data Availability

Sequencing files related to transcriptomic data will be made available on SRA.

## Appendix A Supplementary data

Supplementary Fig. 1 Percent lignin degradation after 10 days A) in the presence and absence of *Phanerochaete chrysosporium* at each tested H_2_O_2_ concentrations combining all iron treatments (N = 25 for each concentration). B) in the presence and absence of *P. ch.* at the tested Fe(II) concentrations combining all H_2_O_2_ concentrations (N = 25 for each concentration). Boxes show interquartile ranges and median values. Supplementary Fig 1

Supplementary Table 1 **Significantly differentially expressed genes, sorted by significance.** Protein ID refers to the protein IDs used by the DOE JGI in the Phanerochaete chrysosporium RP-78 v4.0 genome. LFC is the log2-fold change. Supplementary Table 1

Supplementary Table 2 **Significant “molecular function” gene ontology (GO) terms extracted from differentially expressed genes (DEGs), sorted by significance**. The Frequency, Uniqueness, and Dispensability columns are metrics associated with the removal of redundant GO terms using Revigo: Uniqueness and Dispensability are anticorrelated, and refer to the semantic similarity of GO terms in the Uniprot database. Frequency refers to the frequency of each term in that database. GO terms with Dispensability < 0.25 are displayed in this table. Go terms are sorted by significance, which is assigned using Classic Kolmogorov-Smirnov P values found by comparing the observed distributions of P values to those expected given a null distribution. Supplementary Table 2

Supplementary Table 3 **Significantly enriched KEGG pathways and modules**, determined from the log2-fold changes of the full gene set using clusterProfiler, both sorted by normalized enrichment score (NES). Set size refers to the count of genes within the pathway. P values were adjusted for the false discovery rate using the Benjamin-Hochberg (BH) procedure. Supplementary Table 3

Supplementary Table 4 **Significant differences in lignin degradation between [H**_**2**_**O**_**2**_**] dosing concentrations,** determined by a post hoc Tukey's HSD test at p < 0.05. This test was done across all iron five concentrations, with five replicates for each condition, for a total of 25 samples per group. Lignin degradation at [H_2_O_2_] = 1.5 mM was significantly different from all other [H_2_O_2_] dosing concentrations. Supplementary Table 4

## References

[bb0005] Ai Ming-Qiang, Wang Fang-Fang, Zhang Yu-Zhong, Huang Feng (2014). Purification of pyranose oxidase from the white rot fungus Irpex lacteus and its cooperation with laccase in lignin degradation. Process Biochem..

[bb5000] Alexa A., Rahnenfuhrer J. (2023). topGO: Enrichment Analysis for Gene Ontology. R package.

[bb0010] Aramaki Takuya, Blanc-Mathieu Romain, Endo Hisashi, Ohkubo Koichi, Kanehisa Minoru, Goto Susumu, Ogata Hiroyuki (2020). KofamKOALA: KEGG Ortholog assignment based on profile HMM and adaptive score threshold. Bioinformatics.

[bb0015] Arantes Valdeir, Milagres Adriane M.F., Filley Timothy R., Goodell Barry (2011). Lignocellulosic polysaccharides and lignin degradation by wood decay fungi: the relevance of nonenzymatic Fenton-based reactions. J. Ind. Microbiol. Biotechnol..

[bb0020] Barber Edward A., Liu Ziyi, Smith Stephen R. (2020). Organic contaminant biodegradation by oxidoreductase enzymes in wastewater treatment. Microorganisms.

[bb0025] Bello Mustapha Mohammed, Raman Abdul Aziz Abdul, Asghar Anam (2019). A review on approaches for addressing the limitations of Fenton oxidation for recalcitrant wastewater treatment. Process Saf. Environ. Prot..

[bb0030] Bolger Anthony M., Lohse Marc, Usadel Bjoern (2014). Trimmomatic: a flexible trimmer for Illumina sequence data. Bioinformatics.

[bb0035] Chigu Nomathemba Loice, Hirosue Sinji, Nakamura Chie, Teramoto Hiroshi, Ichinose Hirofumi, Wariishi Hiroyuki (2010). Cytochrome P450 monooxygenases involved in anthracene metabolism by the white-rot basidiomycete Phanerochaete chrysosporium. Appl. Microbiol. Biotechnol..

[bb0040] Coenye Tom (2021). Do results obtained with RNA-sequencing require independent verification?. Biofilms.

[bb0045] Collins P.J., Field J.A., Teunissen P., Dobson A.D. (1997). Stabilization of lignin peroxidases in white rot fungi by tryptophan. Appl. Environ. Microbiol..

[bb0050] Couto Susana Rodríguez, Toca-Herrera José L. (2007). Laccase production at reactor scale by filamentous fungi. Biotechnol. Adv..

[bb0055] Del Álamo A., Cruz M.I., Pariente R. Molina, Martínez F. (2022). Advanced bio-oxidation of fungal mixed cultures immobilized on rotating biological contactors for the removal of pharmaceutical micropollutants in a real hospital wastewater. J. Hazard. Mater..

[bb0060] Del Cerro Carlos, Erickson Erika, Dong Tao, Wong Allison R., Eder Elizabeth K., Purvine Samuel O., Mitchell Hugh D. (2021). Intracellular pathways for lignin catabolism in white-rot fungi. Proc. Natl. Acad. Sci. U. S. A..

[bb0065] Espinosa-Ortiz Erika J., Rene Eldon R., van Hullebusch Eric D., Lens Piet N.L. (2015). Removal of selenite from wastewater in a Phanerochaete chrysosporium pellet based fungal bioreactor. Int. Biodeterior. Biodegradation.

[bb0070] Gómez-Toribio Víctor, García-Martín Ana B., Martínez María J., Martínez Angel T., Guillén Francisco (2009). Induction of extracellular hydroxyl radical production by white-rot fungi through Quinone redox cycling. Appl. Environ. Microbiol..

[bb0075] Harper D.B., McRoberts W.C., Kennedy J.T. (1996). Comparison of the efficacies of chloromethane, methionine, and S-adenosylmethionine as methyl precursors in the biosynthesis of Veratryl alcohol and related compounds in Phanerochaete chrysosporium. Appl. Environ. Microbiol..

[bb0080] Hou Lipeng, Ji Dandan, Dong Weifang, Yuan Lin, Zhang Fengshan, Li Yan, Zang Lihua (2020). The synergistic action of electro-Fenton and white-rot fungi in the degradation of lignin. Front. Bioeng. Biotechnol..

[bb0085] Hu Yuansheng, Hao Xiaodi, Wang Jimin, Cao Yali (2016). Enhancing anaerobic digestion of lignocellulosic materials in excess sludge by bioaugmentation and pre-treatment. Waste Manag..

[bb0090] Janusz Grzegorz, Pawlik Anna, Sulej Justyna, Swiderska-Burek Urszula, Jarosz-Wilkolazka Anna, Paszczynski Andrzej (2017). Lignin degradation: microorganisms, enzymes involved, genomes analysis and evolution. FEMS Microbiol. Rev..

[bb0095] Kameshwar Ayyappa Kumar Sista, Qin Wensheng (2017). Gene expression metadata analysis reveals molecular mechanisms employed by Phanerochaete chrysosporium during lignin degradation and detoxification of plant extractives. Curr. Genet..

[bb0100] Kim Daehwan, Paggi Joseph M., Park Chanhee, Bennett Christopher, Salzberg Steven L. (2019). Graph-based genome alignment and genotyping with HISAT2 and HISAT-genotype. Nat. Biotechnol..

[bb0105] Le Thanh Mai Pham, Kim Yong Hwan (2016). Discovery and characterization of new O-methyltransferase from the genome of the lignin-degrading fungus Phanerochaete chrysosporium for enhanced lignin degradation. Enzym. Microb. Technol..

[bb0110] Liu Jin, Smith Stephen R. (2022). The link between organic matter composition and the biogas yield of full-scale sewage sludge anaerobic digestion. Water Sci. Technol. J. Int. Assoc. Water Pollut. Res..

[bb0115] Liu Shihong, Das Lalitendu, Blauch David N., Veronee Charlie, Dou Chang, Gladden John, Sun Ning, Socha Aaron M. (2020). Statistical design of experiments for production and purification of vanillin and aminophenols from commercial lignin. Green Chem. Int. J. Green Chem. Res. GC.

[bb0120] Marzullo L., Cannio R., Giardina P., Santini M.T., Sannia G. (1995). Veratryl alcohol oxidase from Pleurotus ostreatus participates in lignin biodegradation and prevents polymerization of laccase-oxidized substrates. J. Biol. Chem..

[bb0125] Merino Carolina, Kuzyakov Yakov, Godoy Karina, Cornejo Pablo, Matus Francisco (2020). Synergy effect of peroxidase enzymes and Fenton reactions greatly increase the anaerobic oxidation of soil organic matter. Sci. Rep..

[bb0130] Mir-Tutusaus J.A., Sarrà M., Caminal G. (2016). Continuous treatment of non-sterile hospital wastewater by Trametes versicolor: how to increase fungal viability by means of operational strategies and pretreatments. J. Hazard. Mater..

[bb0135] Mir-Tutusaus Josep Anton, Baccar Rim, Caminal Glòria, Sarrà Montserrat (2018). Can white-rot fungi be a real wastewater treatment alternative for organic micropollutants removal? A review. Water Res..

[bb0140] More T.T., Yan S., Tyagi R.D., Surampalli R.Y. (2010). Potential use of filamentous Fungi for wastewater sludge treatment. Bioresour. Technol..

[bb0145] Moreira M.T., Feijoo G., Lema J.M. (2003). Fungal bioreactors: applications to white-rot Fungi. Rev. Environ. Sci. Biotechnol..

[bb0150] Ohm Robin A., Riley Robert, Salamov Asaf, Min Byoungnam, Choi In-Geol, Grigoriev Igor V. (2014). Genomics of wood-degrading fungi. Fungal Genet. Biol..

[bb0155] Robinson Mark D., McCarthy Davis J., Smyth Gordon K. (2010). edgeR: a bioconductor package for differential expression analysis of digital gene expression data. Bioinformatics.

[bb0160] Samejima M., Eriksson K.E. (1992). A comparison of the catalytic properties of cellobiose:quinone oxidoreductase and cellobiose oxidase from Phanerochaete chrysosporium. Eur. J. Biochem. FEBS.

[bb0165] Sankaran Sindhuja, Khanal Samir Kumar, Jasti Nagapadma, Jin Bo, Pometto Anthony L., Hans Van Leeuwen J. (2010). Use of filamentous fungi for wastewater treatment and production of high value fungal byproducts: a review. Crit. Rev. Environ. Sci. Technol..

[bb0170] Singh Deepak, Chen Shulin (2008). The white-rot fungus Phanerochaete chrysosporium: conditions for the production of lignin-degrading enzymes. Appl. Microbiol. Biotechnol..

[bb0175] Su Yingjie, Xiaoxiao Yu, Sun Yang, Wang Gang, Chen Huan, Chen Guang (2018). Evaluation of screened lignin-degrading Fungi for the biological pretreatment of corn Stover. Sci. Rep..

[bb0180] Supek Fran, Bošnjak Matko, Škunca Nives, Šmuc Tomislav (2011). REVIGO summarizes and visualizes long lists of gene ontology terms. PLoS One.

[bb0185] Syed Khajamohiddin, Doddapaneni Harshavardhan, Subramanian Venkataramanan, Lam Ying Wai, Yadav Jagjit S. (2010). Genome-to-function characterization of novel fungal P450 monooxygenases oxidizing polycyclic aromatic hydrocarbons (PAHs). Biochem. Biophys. Res. Commun..

[bb0190] Teixeira Ricardo S.S., Silva Ayla S., Moutta Rondinele O., Ferreira-Leitão Viridiana S., Barros Rodrigo R.O., Ferrara Maria Antonieta, Bon Elba P.S. (2014). Biomass pretreatment: a critical choice for biomass utilization via biotechnological routes. BMC Proc..

[bb0195] Tien Ming, Kent Kirk T. (1988). Methods in Enzymology.

[bb0200] Wang Jianqiao, Ohno Haruka, Ide Yuuri, Ichinose Hirofumi, Mori Toshio, Kawagishi Hirokazu, Hirai Hirofumi (2019). Identification of the cytochrome P450 involved in the degradation of neonicotinoid insecticide Acetamiprid in Phanerochaete chrysosporium. J. Hazard. Mater..

[bb0205] Wolf Megan E., Hinchen Daniel J., DuBois Jennifer L., McGeehan John E., Eltis Lindsay D. (2022). Cytochromes P450 in the biocatalytic valorization of lignin. Curr. Opin. Biotechnol..

[bb0210] Wu Tianzhi, Hu Erqiang, Xu Shuangbin, Chen Meijun, Guo Pingfan, Dai Zehan, Feng Tingze (2021). clusterProfiler 4.0: a universal enrichment tool for interpreting omics data. Innovation (Cambridge (Mass.)).

[bb0215] Wymelenberg Vanden, Amber Jill Gaskell, Mozuch Mike, Kersten Phil, Sabat Grzegorz, Martinez Diego, Cullen Dan (2009). Transcriptome and Secretome analyses of Phanerochaete Chrysosporium reveal complex patterns of gene expression. Appl. Environ. Microbiol..

[bb0220] Zhang Meng-Hui, Dong Hui, Zhao Liang, Wang De-Xi, Meng Di (2019). A review on Fenton process for organic wastewater treatment based on optimization perspective. Sci. Total Environ..

[bb0225] Zheng Weishuang, Lehmann Anika, Ryo Masahiro, Vályi Kriszta Kezia, Rillig Matthias C. (2020). Growth rate trades off with enzymatic investment in soil filamentous fungi. Sci. Rep..

[bb0230] Zhu Daochen, Zhang Peipei, Xie Changxiao, Zhang Weimin, Sun Jianzhong, Qian Wei-Jun, Yang Bin (2017). Biodegradation of alkaline lignin by Bacillus ligniniphilus L1. Biotechnol. Biofuels.

