# Supplementary material for: Synergistic lignin degradation between Phanerochaete chrysosporium and Fenton chemistry is mediated through iron cycling and ligninolytic enzyme induction
Source: Sci Total Environ. 2023 Dec 20;905:166767. doi: 10.1016/j.scitotenv.2023.166767 (PMC10646785; doi:10.1016/j.scitotenv.2023.166767)
Supplement: Supplementary Fig. 1 — Percent lignin degradation after 10 days A) in the presence and absence of Phanerochaete chrysosporium at each tested H2O2 concentrations combining all iron treatments (N = 25 for each concentration). B) in the presence and absence of P. ch. at the tested Fe(II) concentrations combining all H2O2 concentrations (N = 25 for each concentration). Boxes show interquartile ranges and median values. [file mmc1.docx]

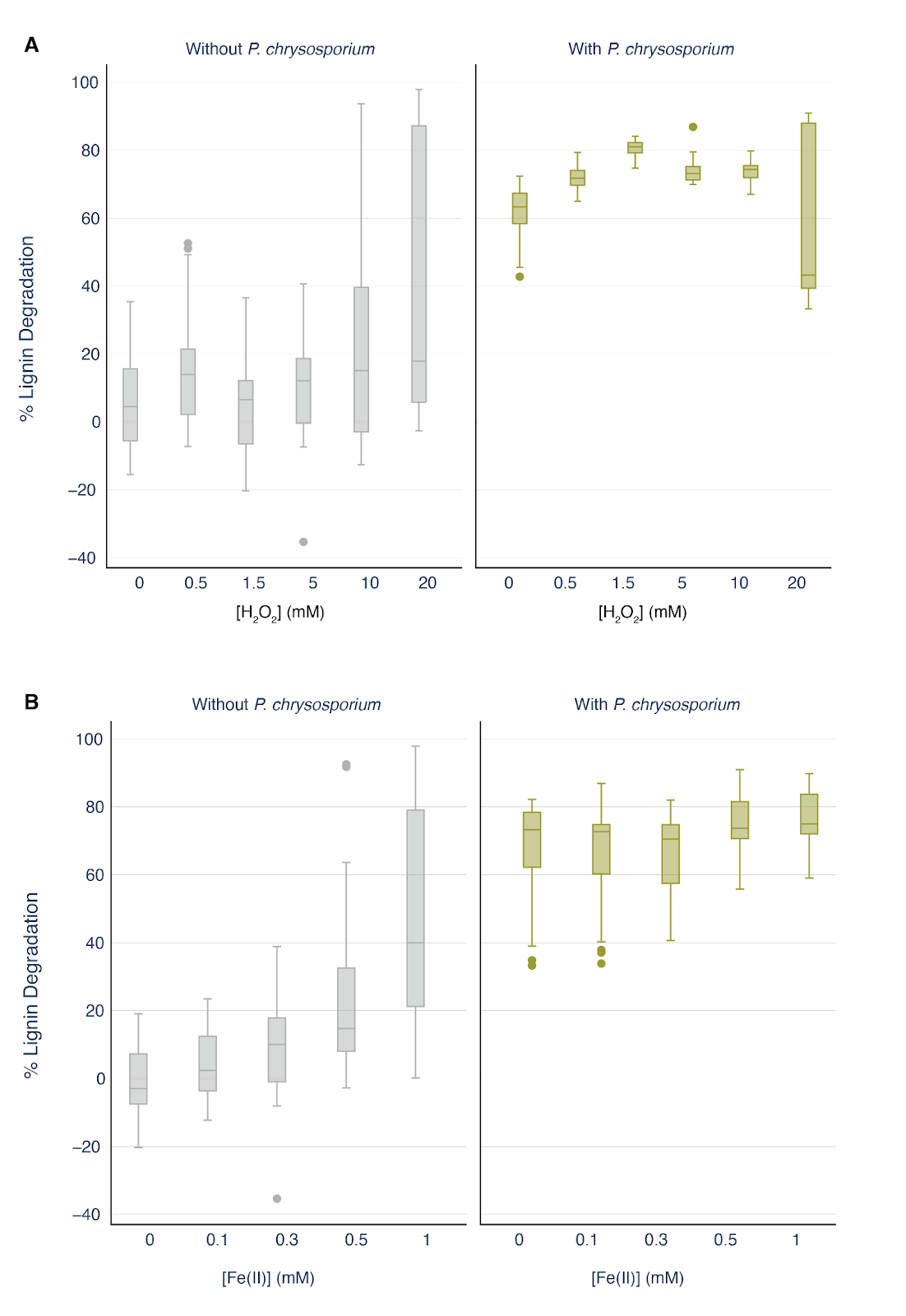


**Percent lignin degradation after 10 days** A) in the presence and absence of *Phanerochaete chrysosporium* at each tested H_2_O_2_ concentrations combining all iron treatments (N = 25 for each concentration). B) in the presence and absence of *P. ch.* at the tested Fe(II) concentrations combining all H_2_O_2_ concentrations (N = 25 for each concentration). Boxes show interquartile ranges and median values.
