# Supplementary material for: Synergistic lignin degradation between Phanerochaete chrysosporium and Fenton chemistry is mediated through iron cycling and ligninolytic enzyme induction
Source: Sci Total Environ. 2023 Dec 20;905:166767. doi: 10.1016/j.scitotenv.2023.166767 (PMC10646785; doi:10.1016/j.scitotenv.2023.166767)
Supplement: Supplementary Table 1 — Significantly differentially expressed genes, sorted by significance. Protein ID refers to the protein IDs used by the DOE JGI in the Phanerochaete chrysosporium RP-78 v4.0 genome. LFC is the log2-fold change. [file mmc2.docx]

**Significantly differentially expressed genes, sorted by significance.** Protein ID refers to the protein IDs used by the DOE JGI in the Phanerochaete chrysosporium RP-78 v4.0 genome. LFC is the log2-fold change.

| **Protein ID** | **LFC** | **FDR** | **Product Name** |
| --- | --- | --- | --- |
| 6390646 | 7.21 | 6.24E-04 | FAD dependent oxidoreductase |
| 6124111 | 6.93 | 3.90E-04 | sulfurtransferase protein |
| 6387663 | 6.44 | 1.66E-03 | acetate--CoA ligase |
| 5318734 | 5.36 | 3.34E-04 | acetyl-CoA synthetase-like protein |
| 6115212 | 5.07 | 6.33E-06 | Acetyltransferase (GNAT) domain containing protein |
| 6200092 | 5.07 | 1.84E-03 | AAA family ATPase |
| 6200172 | 4.60 | 2.08E-04 | AAA family ATPase |
| 6199153 | 4.27 | 9.21E-06 | GNAT family N-acetyltransferase |
| 6199370 | 4.11 | 1.59E-03 | GNAT family N-acetyltransferase |
| 6328988 | 4.10 | 2.52E-04 | NAD(P)-binding protein |
| 6295626 | 4.05 | 7.90E-05 | S-adenosyl-L-methionine-dependent methyltransferase |
| 6249462 | 3.94 | 9.23E-05 | uncharacterized protein PHACADRAFT_250218 |
| 6214880 | 3.91 | 8.44E-03 | phosphoadenosine phosphosulfate reductase family protein |
| 6200336 | 3.90 | 2.78E-04 | ATP-binding protein |
| 6292697 | 3.83 | 5.28E-03 | hypothetical protein PsYK624_074920 |
| 6271350 | 3.79 | 7.77E-06 | hypothetical protein |
| 5813411 | 3.67 | 3.20E-03 | acetate--CoA ligase |
| 6363782 | 3.66 | 1.57E-04 | hypothetical protein |
| 6200353 | 3.63 | 5.95E-04 | ATP-binding protein |
| 6288403 | 3.56 | 2.37E-04 | kinesin motor domain-containing protein |
| 6232356 | 3.52 | 5.71E-05 | cytochrome P450 |
| 6222191 | 3.51 | 3.33E-04 | uncharacterized protein PHACADRAFT_257636 |
| 6104383 | 3.47 | 8.00E-04 | hypothetical protein PsYK624_070780 |
| 6247992 | 3.42 | 3.23E-03 | hypothetical protein PsYK624_007050 |
| 6291401 | 3.37 | 2.69E-03 | tyrosine ammonia-lyase |
| 6416945 | 3.36 | 7.68E-04 | questin oxidase family protein |
| 4161660 | 3.34 | 7.37E-06 | Zn(II)2Cys6 transcription factor |
| 6121022 | 3.30 | 1.07E-03 | von Willebrand factor type A domain-domain containing protein |
| 4466665 | 3.29 | 3.98E-03 | PLP-dependent transferase |
| 4027379 | 3.27 | 2.82E-04 | Isochorismatase hydrolase |
| 6201380 | 3.27 | 7.29E-05 | aldehyde dehydrogenase |
| 6108199 | 3.26 | 6.74E-03 | FAD dependent oxidoreductase |
| 6370042 | 3.20 | 2.12E-04 | LysM peptidoglycan-binding domain-containing protein |
| 6358133 | 3.16 | 1.12E-03 | multidrug resistance-associated ABC transporter |
| 6320820 | 3.11 | 6.33E-06 | aquaporin |
| 6199299 | 3.07 | 2.35E-03 | S-adenosyl-L-methionine-dependent methyltransferase |
| 6228712 | 2.93 | 4.81E-06 | sulfite oxidase |
| 6240942 | 2.92 | 4.68E-04 | expressed protein |
| 6248227 | 2.92 | 2.07E-03 | MFS general substrate transporter |
| 6292889 | 2.87 | 1.28E-03 | NADP-dependent oxidoreductase |
| 6335850 | 2.79 | 1.05E-03 | hypothetical protein PsYK624_013220 |
| 6303089 | 2.69 | 8.38E-04 | mitochondrial amino-acid acetyltransferase |
| 6339174 | 2.59 | 5.51E-03 | proline-specific peptidase |
| 6351897 | 2.56 | 2.17E-03 | cytochrome P450 |
| 6326664 | 2.56 | 8.44E-03 | GNAT family N-acetyltransferase |
| 6107466 | 2.55 | 2.40E-03 | PARP-domain-containing protein |
| 6373469 | 2.55 | 1.54E-03 | snf2 superfamily protein |
| 6326455 | 2.52 | 4.49E-03 | hypothetical protein PsYK624_079920 |
| 6351454 | 2.48 | 1.57E-03 | P-loop containing nucleoside triphosphate hydrolase protein |
| 6319045 | 2.48 | 2.35E-03 | 54S ribosomal protein L16, mitochondrial |
| 6217883 | 2.47 | 5.70E-03 | aldehyde reductase |
| 6269212 | 2.46 | 8.14E-03 | hypothetical protein PsYK624_032720 |
| 6337416 | 2.46 | 6.26E-04 | hypothetical protein |
| 6277905 | 2.45 | 2.81E-04 | glutathione reductase |
| 6272497 | 2.43 | 5.58E-06 | NAD(P)-dependent alcohol dehydrogenase |
| 6387317 | 2.43 | 8.70E-04 | Zn(II)2Cys6 transcription factor |
| 6262041 | 2.40 | 3.85E-03 | hypothetical protein PsYK624_068350 |
| 6226166 | 2.37 | 1.04E-03 | cytochrome P450 |
| 6472013 | 2.34 | 3.20E-03 | MFS general substrate transporter |
| 6373918 | 2.27 | 7.13E-03 | aldehyde dehydrogenase family protein |
| 4383439 | 2.26 | 8.39E-03 | expressed protein |
| 6325584 | 2.16 | 2.56E-03 | cytochrome P450 |
| 6277542 | 2.14 | 2.35E-03 | flavin-containing monooxygenase family protein |
| 6260313 | 2.14 | 8.12E-03 | predicted protein |
| 6319287 | 2.10 | 6.33E-06 | pepsin-like aspartic proteases |
| 6298884 | 2.09 | 3.20E-03 | multidrug resistance-associated ABC transporter |
| 6298828 | 2.04 | 1.07E-03 | multidrug resistance-associated ABC transporter |
| 6359683 | 2.01 | 1.21E-03 | NADH dehydrogenase-like protein |
| 6200442 | 2.00 | 4.68E-03 | alpha-ketoglutarate-dependent dioxygenase AlkB |
| 6321639 | -2.00 | 8.44E-03 | isocitrate lyase |
| 4011688 | -2.03 | 1.29E-04 | NCS1 nucleoside transporter family |
| 6223205 | -2.04 | 2.55E-05 | hypothetical protein PsYK624_120780 |
| 6256187 | -2.05 | 3.07E-03 | hexose transporter |
| 6340099 | -2.15 | 6.21E-04 | hypothetical protein |
| 6381863 | -2.16 | 6.91E-06 | aldo/keto reductase |
| 6333053 | -2.17 | 8.40E-07 | thiamine diphosphate-binding protein |
| 6353535 | -2.22 | 1.66E-04 | MFS sugar transporter |
| 6281592 | -2.33 | 1.42E-04 | aldo/keto reductase |
| 6283694 | -2.49 | 1.42E-04 | zinc-binding alcohol dehydrogenase family protein |
| 6355975 | -2.52 | 1.13E-04 | uncharacterized protein PHACADRAFT_254135 |
| 6286697 | -2.57 | 6.92E-03 | fungal hydrophobin |
| 6321836 | -2.73 | 1.46E-07 | MFS general substrate transporter |
| 6282962 | -3.54 | 3.83E-15 | medium-chain dehydrogenase/reductase like protein |
