# Supplementary material for: Synergistic lignin degradation between Phanerochaete chrysosporium and Fenton chemistry is mediated through iron cycling and ligninolytic enzyme induction
Source: Sci Total Environ. 2023 Dec 20;905:166767. doi: 10.1016/j.scitotenv.2023.166767 (PMC10646785; doi:10.1016/j.scitotenv.2023.166767)
Supplement: Supplementary Table 2 — Significant “molecular function” gene ontology (GO) terms extracted from differentially expressed genes (DEGs), sorted by significance. The Frequency, Uniqueness, and Dispensability columns are metrics associated with the removal of redundant GO terms using Revigo: Uniqueness and Dispensability are anticorrelated, and refer to the semantic similarity of GO terms in the Uniprot database. Frequency refers to the frequency of each term in that database. GO terms with Dispensability < 0.25 are displayed in this table. Go terms are sorted by significance, which is assigned using Classic Kolmogorov-Smirnov P values found by comparing the observed distributions of P values to those expected given a null distribution. [file mmc3.docx]

**Significant “molecular function” gene ontology (GO) terms extracted from differentially expressed genes (DEGs), sorted by significance**. The Frequency, Uniqueness, and Dispensability columns are metrics associated with the removal of redundant GO terms using Revigo: Uniqueness and Dispensability are anticorrelated, and refer to the semantic similarity of GO terms in the Uniprot database. Frequency refers to the frequency of each term in that database. GO terms with Dispensability < 0.25 are displayed in this table. Go terms are sorted by significance, which is assigned using Classic Kolmogorov-Smirnov P values found by comparing the observed distributions of P values to those expected given a null distribution.

| **GO-term ID** | **Description** | **Frequency** | **Uniqueness** | **Dispensability** | **Classic KS**  **P values** |
| --- | --- | --- | --- | --- | --- |
| GO:0016491 | oxidoreductase activity | 12.06 | 0.93 | 0.05 | 3.00E-11 |
| GO:0016627 | oxidoreductase activity, acting on the CH-CH group of donors | 0.95 | 0.82 | 0.00 | 2.00E-06 |
| GO:0005506 | iron ion binding | 1.46 | 0.98 | 0.00 | 9.60E-06 |
| GO:0016614 | oxidoreductase activity, acting on CH-OH group of donors | 1.61 | 0.82 | 0.23 | 1.50E-05 |
| GO:0016616 | oxidoreductase activity, acting on the CH-OH group of donors, NAD or NADP as acceptor | 1.37 | 0.75 | 0.23 | 3.40E-05 |
| GO:0003824 | catalytic activity | 62.15 | 1.00 | 0.00 | 1.40E-04 |
| GO:0016705 | oxidoreductase activity, acting on paired donors, with incorporation or reduction of molecular oxygen | 1.30 | 0.82 | 0.23 | 2.00E-04 |
| GO:0004190 | aspartic-type endopeptidase activity | 0.27 | 0.89 | 0.05 | 3.90E-04 |
| GO:0046914 | transition metal ion binding | 5.90 | 0.98 | 0.19 | 4.50E-04 |
| GO:0015144 | carbohydrate transmembrane transporter activity | 0.23 | 0.88 | 0.00 | 4.80E-04 |
| GO:0020037 | heme binding | 1.43 | 0.98 | 0.06 | 9.10E-04 |
| GO:0046906 | tetrapyrrole binding | 1.61 | 0.98 | 0.10 | 9.10E-04 |
| GO:0043169 | cation binding | 18.10 | 0.98 | 0.12 | 1.38E-03 |
| GO:0016675 | oxidoreductase activity, acting on a heme group of donors | 0.15 | 0.82 | 0.23 | 1.46E-03 |
| GO:0046872 | metal ion binding | 17.93 | 0.98 | 0.19 | 1.59E-03 |
| GO:0050661 | NADP binding | 0.63 | 0.98 | 0.10 | 2.33E-03 |
| GO:0033765 | steroid dehydrogenase activity, acting on the CH-CH group of donors | 0.03 | 0.80 | 0.23 | 8.19E-03 |
| GO:0016829 | lyase activity | 3.57 | 0.93 | 0.05 | 1.09E-02 |
| GO:0003684 | damaged DNA binding | 0.25 | 0.98 | 0.10 | 1.25E-02 |
| GO:0004551 | nucleotide diphosphatase activity | 0.07 | 0.88 | 0.18 | 1.41E-02 |
| GO:0008094 | ATP-dependent activity, acting on DNA | 1.08 | 0.90 | 0.05 | 1.63E-02 |
| GO:0016701 | oxidoreductase activity, acting on single donors with incorporation of molecular oxygen | 0.30 | 0.82 | 0.23 | 2.01E-02 |
| GO:0071949 | FAD binding | 0.56 | 0.97 | 0.18 | 2.20E-02 |
| GO:0000981 | DNA-binding transcription factor activity, RNA polymerase II-specific | 0.88 | 1.00 | 0.00 | 2.26E-02 |
| GO:0004553 | hydrolase activity, hydrolyzing O-glycosyl compounds | 1.57 | 0.90 | 0.18 | 2.54E-02 |
| GO:0016830 | carbon-carbon lyase activity | 1.03 | 0.93 | 0.05 | 2.57E-02 |
| GO:0016798 | hydrolase activity, acting on glycosyl bonds | 2.00 | 0.90 | 0.18 | 2.76E-02 |
| GO:0008168 | methyltransferase activity | 2.79 | 0.92 | 0.05 | 2.90E-02 |
| GO:0016741 | transferase activity, transferring one-carbon groups | 3.05 | 0.93 | 0.17 | 3.16E-02 |
| GO:0003960 | NADPH:quinone reductase activity | 0.01 | 0.82 | 0.23 | 3.44E-02 |
| GO:0016810 | hydrolase activity, acting on carbon-nitrogen (but not peptide) bonds | 1.67 | 0.90 | 0.18 | 3.51E-02 |
| GO:0016229 | steroid dehydrogenase activity | 0.07 | 0.82 | 0.23 | 4.05E-02 |
| GO:0004497 | monooxygenase activity | 1.21 | 0.82 | 0.23 | 4.18E-02 |
| GO:0051213 | dioxygenase activity | 0.63 | 0.82 | 0.23 | 4.21E-02 |
| GO:0016879 | ligase activity, forming carbon-nitrogen bonds | 1.22 | 0.93 | 0.05 | 4.30E-02 |
