# Supplementary material for: Synergistic lignin degradation between Phanerochaete chrysosporium and Fenton chemistry is mediated through iron cycling and ligninolytic enzyme induction
Source: Sci Total Environ. 2023 Dec 20;905:166767. doi: 10.1016/j.scitotenv.2023.166767 (PMC10646785; doi:10.1016/j.scitotenv.2023.166767)
Supplement: Supplementary Table 3 — Significantly enriched KEGG pathways and modules, determined from the log2-fold changes of the full gene set using clusterProfiler, both sorted by normalized enrichment score (NES). Set size refers to the count of genes within the pathway. P values were adjusted for the false discovery rate using the Benjamin-Hochberg (BH) procedure. [file mmc4.docx]

**Significantly enriched KEGG pathways and modules**, determined from the log2-fold changes of the full gene set using clusterProfiler, both sorted by normalized enrichment score (NES). Set size refers to the count of genes within the pathway. P values were adjusted for the false discovery rate using the Benjamin-Hochberg (BH) procedure.

| **KEGG ID** | **Description** | **Set size** | **NES** | **FDR-adjusted P-value** |
| --- | --- | --- | --- | --- |
| ko03008 | Ribosome biogenesis in eukaryotes | 62 | 2.262 | 1.53E-05 |
| ko01210 | 2-Oxocarboxylic acid metabolism | 30 | 2.253 | 1.24E-04 |
| ko00300 | Lysine biosynthesis | 13 | 2.153 | 1.24E-04 |
| ko01230 | Biosynthesis of amino acids | 108 | 2.137 | 1.53E-05 |
| ko00400 | Phenylalanine, tyrosine and tryptophan biosynthesis | 24 | 1.993 | 1.93E-03 |
| ko00330 | Arginine and proline metabolism | 25 | 1.937 | 1.01E-02 |
| ko00290 | Valine, leucine and isoleucine biosynthesis | 12 | 1.925 | 1.90E-02 |
| ko01110 | Biosynthesis of secondary metabolites | 335 | 1.912 | 7.47E-07 |
| ko00220 | Arginine biosynthesis | 19 | 1.871 | 3.49E-02 |
| ko04915 | Estrogen signaling pathway | 10 | 1.825 | 4.33E-02 |
| ko03013 | Nucleocytoplasmic transport | 63 | 1.804 | 2.28E-02 |
| ko03015 | mRNA surveillance pathway | 48 | 1.785 | 4.21E-02 |
| ko00643 | Styrene degradation | 4 | 1.745 | 1.50E-02 |
| ko04141 | Protein processing in endoplasmic reticulum | 77 | 1.671 | 4.92E-02 |
| ko00940 | Phenylpropanoid biosynthesis | 3 | 1.627 | 4.92E-02 |
| ko01250 | Biosynthesis of nucleotide sugars | 23 | -1.808 | 4.77E-02 |
| ko00710 | Carbon fixation in photosynthetic organisms | 15 | -1.912 | 3.08E-02 |
| ko00190 | Oxidative phosphorylation | 92 | -1.981 | 2.72E-04 |
| ko00051 | Fructose and mannose metabolism | 20 | -2.019 | 1.01E-02 |
| ko00040 | Pentose and glucuronate interconversions | 21 | -2.026 | 1.01E-02 |

| **KEGG ID** | **Description** | **Set size** | **NES** | **FDR-adjusted P-value** |
| --- | --- | --- | --- | --- |
| M00023 | Tryptophan biosynthesis, chorismate => tryptophan | 10 | 2.042 | 1.91E-03 |
| M00030 | Lysine biosynthesis, AAA pathway, 2-oxoglutarate => 2-aminoadipate => lysine | 9 | 1.998 | 1.91E-03 |
| M00051 | De novo pyrimidine biosynthesis, glutamine (+ PRPP) => UMP | 10 | 1.882 | 3.09E-02 |
| M00039 | Monolignol biosynthesis, phenylalanine/tyrosine => monolignol | 3 | 1.546 | 3.33E-02 |
| M00344 | Formaldehyde assimilation, xylulose monophosphate pathway | 3 | -1.647 | 3.10E-02 |
| M00014 | Glucuronate pathway (uronate pathway) | 5 | -1.813 | 3.21E-02 |
| M00165 | Reductive pentose phosphate cycle (Calvin cycle) | 6 | -1.842 | 3.10E-02 |
| M00002 | Glycolysis, core module involving three-carbon compounds | 7 | -1.849 | 3.09E-02 |
| M00001 | Glycolysis (Embden-Meyerhof pathway), glucose => pyruvate | 11 | -1.964 | 2.85E-02 |
| M00003 | Gluconeogenesis, oxaloacetate => fructose-6P | 9 | -2.063 | 6.91E-03 |

| **KEGG Pathway enrichment** | | | | |
| --- | --- | --- | --- | --- |
| **KEGG ID** | **Description** | **Set size** | **NES** | **FDR-adjusted P-value** |
| ko03008 | Ribosome biogenesis in eukaryotes | 62 | 2.262 | 1.53E-05 |
| ko01210 | 2-Oxocarboxylic acid metabolism | 30 | 2.253 | 1.24E-04 |
| ko00300 | Lysine biosynthesis | 13 | 2.153 | 1.24E-04 |
| ko01230 | Biosynthesis of amino acids | 108 | 2.137 | 1.53E-05 |
| ko00400 | Phenylalanine, tyrosine and tryptophan biosynthesis | 24 | 1.993 | 1.93E-03 |
| ko00330 | Arginine and proline metabolism | 25 | 1.937 | 1.01E-02 |
| ko00290 | Valine, leucine and isoleucine biosynthesis | 12 | 1.925 | 1.90E-02 |
| ko01110 | Biosynthesis of secondary metabolites | 335 | 1.912 | 7.47E-07 |
| ko00220 | Arginine biosynthesis | 19 | 1.871 | 3.49E-02 |
| ko04915 | Estrogen signaling pathway | 10 | 1.825 | 4.33E-02 |
| ko03013 | Nucleocytoplasmic transport | 63 | 1.804 | 2.28E-02 |
| ko03015 | mRNA surveillance pathway | 48 | 1.785 | 4.21E-02 |
| ko00643 | Styrene degradation | 4 | 1.745 | 1.50E-02 |
| ko04141 | Protein processing in endoplasmic reticulum | 77 | 1.671 | 4.92E-02 |
| ko00940 | Phenylpropanoid biosynthesis | 3 | 1.627 | 4.92E-02 |
| ko01250 | Biosynthesis of nucleotide sugars | 23 | -1.808 | 4.77E-02 |
| ko00710 | Carbon fixation in photosynthetic organisms | 15 | -1.912 | 3.08E-02 |
| ko00190 | Oxidative phosphorylation | 92 | -1.981 | 2.72E-04 |
| ko00051 | Fructose and mannose metabolism | 20 | -2.019 | 1.01E-02 |
| ko00040 | Pentose and glucuronate interconversions | 21 | -2.026 | 1.01E-02 |
| **KEGG Module Enrichment** | | | | |
| **KEGG ID** | **Description** | **Set size** | **NES** | **FDR-adjusted P-value** |
| M00023 | Tryptophan biosynthesis, chorismate => tryptophan | 10 | 2.042 | 1.91E-03 |
| M00030 | Lysine biosynthesis, AAA pathway, 2-oxoglutarate => 2-aminoadipate => lysine | 9 | 1.998 | 1.91E-03 |
| M00051 | De novo pyrimidine biosynthesis, glutamine (+ PRPP) => UMP | 10 | 1.882 | 3.09E-02 |
| M00039 | Monolignol biosynthesis, phenylalanine/tyrosine => monolignol | 3 | 1.546 | 3.33E-02 |
| M00344 | Formaldehyde assimilation, xylulose monophosphate pathway | 3 | -1.647 | 3.10E-02 |
| M00014 | Glucuronate pathway (uronate pathway) | 5 | -1.813 | 3.21E-02 |
| M00165 | Reductive pentose phosphate cycle (Calvin cycle) | 6 | -1.842 | 3.10E-02 |
| M00002 | Glycolysis, core module involving three-carbon compounds | 7 | -1.849 | 3.09E-02 |
| M00001 | Glycolysis (Embden-Meyerhof pathway), glucose => pyruvate | 11 | -1.964 | 2.85E-02 |
| M00003 | Gluconeogenesis, oxaloacetate => fructose-6P | 9 | -2.063 | 6.91E-03 |
