# Supplementary material for: Synergistic lignin degradation between Phanerochaete chrysosporium and Fenton chemistry is mediated through iron cycling and ligninolytic enzyme induction
Source: Sci Total Environ. 2023 Dec 20;905:166767. doi: 10.1016/j.scitotenv.2023.166767 (PMC10646785; doi:10.1016/j.scitotenv.2023.166767)
Supplement: Supplementary Table 4 — Significant differences in lignin degradation between [H2O2] dosing concentrations, determined by a post hoc Tukey's HSD test at p < 0.05. This test was done across all iron five concentrations, with five replicates for each condition, for a total of 25 samples per group. Lignin degradation at [H2O2] = 1.5 mM was significantly different from all other [H2O2] dosing concentrations. [file mmc5.docx]

**Significant differences in lignin degradation between [H_2_O_2_] dosing concentrations,** determined by a post hoc Tukey’s HSD test at p<0.05. This test was done across all iron five concentrations, with five replicates for each condition, for a total of 25 samples per group. Lignin degradation at [H_2_O_2_]=1.5 mM was significantly different from all other [H_2_O_2_] dosing concentrations.

| [H_2_O_2_] group 1 | [H_2_O_2_] group 2 | **Difference** | **Lower** | **Upper** | **q-value** | **p-value** |
| --- | --- | --- | --- | --- | --- | --- |
| 0.5 | 0 | 9.660432 | 5.960713 | 13.36015 | 10.2361 | <0.001 |
| 1.5 | 0 | 18.5049 | 14.77108 | 22.23872 | 19.42854 | <0.001 |
| 5 | 0 | 11.76604 | 8.066322 | 15.46576 | 12.46718 | <0.001 |
| 10 | 0 | 11.75959 | 8.059866 | 15.4593 | 12.46034 | <0.001 |
| 1.5 | 0.5 | 8.844468 | 5.320192 | 12.36874 | 9.838037 | <0.001 |
| 1.5 | 5 | 6.738859 | 3.214583 | 10.26314 | 7.495888 | <0.001 |
| 1.5 | 10 | 6.745315 | 3.221039 | 10.26959 | 7.503069 | <0.001 |
